# Perioperative and long-term operative outcomes after surgery for trigeminal neuralgia: microvascular decompression vs percutaneous balloon ablation

**DOI:** 10.1186/1746-160X-4-11

**Published:** 2008-07-02

**Authors:** W Scott Jellish, William Benedict, Kevin Owen, Douglas Anderson, Elaine Fluder, John F Shea

**Affiliations:** 1 Department of Anesthesiology, Loyola University Medical Center, Maywood, IL, USA; 2 Department of Neurosurgery, Loyola University Medical Center, Maywood, IL, USA

## Abstract

**Objectives:**

Numerous medical and surgical therapies have been utilized to treat the symptoms of trigeminal neuralgia (TN). This retrospective study compares patients undergoing either microvascular decompression or balloon ablation of the trigeminal ganglion and determines which produces the best long-term outcomes.

**Methods:**

A 10-year retrospective chart review was performed on patients who underwent microvascular decompression (MVD) or percutaneous balloon ablation (BA) surgery for TN. Demographic data, intraoperative variables, length of hospitalization and symptom improvement were assessed along with complications and recurrences of symptoms after surgery. Appropriate statistical comparisons were utilized to assess differences between the two surgical techniques.

**Results:**

MVD patients were younger but were otherwise similar to BA patients. Intraoperatively, twice as many BA patients developed bradycardia compared to MVD patients. 75% of BA patients with bradycardia had an improvement of symptoms. Hospital stay was shorter in BA patients but overall improvement of symptoms was better with MVD. Postoperative complication rates were similar (21% vs 26%) between the BA and MVD groups.

**Discussion:**

MVD produced better overall outcomes compared to BA and may be the procedure of choice for surgery to treat TN.

## Background

Trigeminal Neuralgia (TN) is "a sudden brief, usually unilateral, severe, recurrent pain in the distribution of one or more branches of the fifth cranial nerve" [1]. This pain is typically triggered by daily activities such as eating, talking, or brushing teeth. Frequently patients are asymptomatic between episodes. Although rare, affecting approximately 4 per 100,000 persons per year, this severe chronic pain syndrome can greatly compromise patient quality of life and disrupt daily functioning [2]. The etiology of trigeminal neuralgia in the majority of cases is compression of the nerve root by a blood vessel [3]. Other, albeit less common etiologies include demylelinating processes such as Multiple Sclerosis (MS), posterior fossa meningiomas or neuromas [3].

Pharmacotherapy is generally the mainstay of treatment of TN, with carbamezapine affording a satisfactory initial effect in approximately 70% of patients [3]. Other medications such as gabapentin, baclofen, oxcarbazepine, and lamotrigine have also been used as primary treatments or as adjuvants to carbamazepine as well [3]. However, loss of pharmacological effect or problems with tolerability of the medications is experienced in almost half of patients by 10 years of treatment [3]. Fortunately, if medical treatment fails then surgical options are available.

Minimally invasive percutaneous techniques include radiofrequency rhizotomy, glycerol rhizotomy, and balloon compression gangliolysis (BA) [4]. More invasive techniques such as posterior fossa exploration for microvascular decompression or partial trigeminal rhizotomy can also be performed [5]. Microvascular decompression (MVD) is currently the only technique which corrects the hypothesized vascular etiology by repositioning the impinging vessel, usually the superior cerebellar artery or the anterior inferior cerebellar artery [6]. Multiple prior comparisons between ablative procedures and MVD exist in the literature but no recent analysis has compared BA and MVD [6-10].

Traditionally, BA is reserved for older patients or patients who may not be able to endure a craniotomy for MVD. However, it is often surgeon preference that may influence what surgical technique the patient will undergo. While both techniques have unique advantages, these procedures are not without complications or side effects. Manoru, et al. first described a trigeminal depressor response (TDR) in which bradycardia occurred as a result of stimulation of the spinal trigeminal complex in rabbits [11]. This TDR ensued after low frequency stimulation of any branch of the trigeminal nerve or entering roots. In another study, Preul, et al. observed bradycardia in 30% of rabbits receiving percutaneous balloon compression of the trigeminal ganglion as well as histopathological changes indicating cellular injury near the inflated balloon [12]. In humans, Brown and Preul described a similar depressor response with percutaneous microcompression of the trigeminal ganglion for the relief of trigeminal neuralgia [13].

Although other investigators have explored the incidence of intraoperative bradycardia with various ablative procedures, none have yet addressed the question of whether or not the occurrence of bradycardia may be used as an indirect indicator of sufficient neuronal injury to predict a successful outcome of the ablative procedure [13,14].

This paper compares the degree of pain relief and occurrence of complications between MVD and BA. It also examines the incidence of intraoperative bradycardia, an indicator of trigeminal stimulation with therapeutic outcomes and attempts to determine which procedure produces the best therapeutic benefit for patients with TN.

## Methods

After obtaining approval from the institutional review board to perform a retrospective chart review, records of patients who had trigeminal neuralgia and underwent either a microvascular decompression via a lateral skull base approach or a balloon compression rhizolysis of the trigeminal ganglion between 1993 and 2003 were reviewed. 120 patients had a total of 164 surgeries during the review period. Cases of atypical neuralgia, carcinomatous pain or multiple sclerosis were excluded. All patients had undergone conservative treatment prior to surgical intervention.

Patients undergoing percutaneous balloon compression (84 cases) had general anesthesia with either a tracheal intubation or placement of laryngeal mask airway. Patients were placed in a supine position with the neck and thorax slightly flexed. Using biplane fluoroscopy, a Trucut liver biopsy needle was inserted into the foramen ovale. A ventricular stylet was next inserted into the hub of the needle. The catheter was then pushed up to the foramen ovale. A number 4 Fogerty catheter was placed into Meckel's Cave and the balloon inflated with 0.75 cc of contrast media for 3 minutes. The presence of a pear-shape was seen when compression of the nerve was accomplished with the balloon. Reflex bradycardia, tearing of the eye, and a modest hypertension insured proper placement.

Microvascular decompression (80 cases) was done under general anesthesia. Invasive blood pressure monitoring was used in a majority of these cases. The patient's head was turned contralaterally and a small retrosigmoid craniotomy was performed. The trigeminal nerve was examined microsurgically for vascular compression at or near its point of entry into the brain stem. Compressive arteries and veins were repositioned and any other compressive veins were electrocoagulated or divided. In some patients a rhizotomy was also performed. These patients had atypical symptoms which were severe enough to warrant immediate relief with no response to carbamazepine. Other criteria for performing a rhizotomy included any patient who had previous invasive procedures for pain relief or where actual vascular compressive findings during surgery were not severe enough to account for the patient's symptoms.

Demographic data was obtained from the records and the length of disease recorded. Pain medications and previous treatments for TN were also recorded. Intraoperative variables collected include OR surgical times, performance of surgical rhizotomy, incidence of bradycardia during treatment (defined as a decrease of 10% from baseline) and hospital length of a stay after the procedure. Postoperative outcomes including the number of patients with immediate improvement in symptoms, percent of total cure and the number with no change in symptoms were also obtained along with the length of time to follow up and the number of patients who relapsed. Time to relapse was recorded, as was the total number of operations performed. Reported complications after surgery were also compared between the two surgical groups and the incidence of the most frequent complications were compared between the two surgical populations. In addition, the incidence of bradycardia was correlated to determine if any relationship exists between the degree of bradycardia and improvement of systems. Numeric variables were examined and compared using two tailed t test analysis while nonparametric variables were compared by Pearson Chi Square Analysis with a p < 0.05 considered significant. All values are expressed as mean ± SEM.

## Results

A total of 84 patients were in the BA group, while the MVD group had 80 patients. All MVDs were performed by one surgeon (DA), while all BAs were performed by another surgeon (JS). Patients who underwent MVD were significantly younger than BA patients (Table 1). Gender, American Society of Anesthesiologists (ASA) physical status, and length of treatment for TN was approximately the same in both groups, and a similar utilization of preoperative medications to control TN symptoms were used (Table 1). A significantly greater number of patients who received trigger point injections underwent BA treatment compared to patients who had MVD (Table 1). Finally, both groups had a similar incidence of prior surgeries for correction of TN (Table 1).

**Table 1:** Demographic Data

	**BA**	**MVD**
Age (years)	66.1 ± 1.5	55.6 ± 1.6 *
Gender (%)		
Male	33(39%)	37(46%)
Female	51(61%)	43(54%)
ASA Physical Status (%)		
1	8	13
2	69	67
3	20	19
4	2	1
Disease Length (Years)	7.0 ± 0.8 (0.3 – 40)	5.3 ± 0.7 (0.3 – 32)
Number of Medications **		
0	7	1
1	48	43
2	36	38
3 or >	11	19
Incidence of Pre-operative Trigger Point Injections (%)	13.1	3.8 *
Incidence of prior surgery for TN (%)	26	28

* P < 0.05 compared to BA

All data reported as mean ± SEM, ** Medications use perioperatively include: Carbemazepine, Phenytoin, Baclofen, Neurontin, Depakote) used pre-operatively (%)

As would be expected, MVD OR time was significantly longer than in the BA group (Table 2). Patients who underwent additional rhizotomy had OR times similar to MVD. A significantly greater number of patients developed bradycardia after BA compared to those who underwent MVD or MVD with rhizotomy (Table 2). In addition, significantly more BA patients developed bradycardia that was severe enough to require treatment.

**Table 2:** Intraoperative Variables and Incidence of Bradycardia

	**BA**	**MVD**	**MVD with Rhizotomy**	**Combined**
Total Operating times (min)	23 ± 2	186 ± 6 *	197 ± 7 *	192 ± 5*
Bradycardia	64/84 (76%)	16/37 (43%) *	15/43 (35%)*	31/80 (39%)
Bradycardia Requiring Treatment	16/84 (19%)	0*	3/43 (7%)	33/80 (4%)*

* P < 0.05 compared to BA group

All data reported as mean ± SEM

Combined = MVD with and without rhizotomy

Bradycardia = 10% or greater decrease in Heart Rate

Length of stay was shorter with percutaneous BA compared with MVD (Table 3). MVD produced a significantly greater number of patients with an immediate improvement of symptoms, as well as cure rate (defined as complete resolution of symptoms at last documented follow-up) (Table 3). A smaller number of MVD compared to BA patients (11% vs 27%) had no improvement after surgery (Table 3). All patients who developed bradycardia during BA had an improvement of symptoms. Similar improvements in symptoms were noted in MVD and MVD with rhizotomy patients who developed bradycardia during the procedure (Table 3). While the incidence of relapse after surgery was the same in both groups, the length of time to relapse was slightly shorter after MVD as compared to BA. However, the requirement for postoperative pharmacologic therapy to treat TN was significantly reduced after MVD (Table 3).

**Table 3:** Postoperative Outcomes for Rhizotomy, Balloon Ablation, and MVD

	**BA**	**MVD**	**Rhizotomy**	**Combined**
Hospital Length of Stay (Days)	2.6 ± 0.4	5.7 ± 0.6 *	4.3 ± 0.2	5.0 ± 0.3
Without Complications (Days)	0.5 ± 0.1	4.6 ± 0.6	3.3 ± 0.3	
Length of Follow-up (Months)	21.8 ± 3.2	33.4 ± 7.1	21.0 ± 4.9	26.5 ± 42
Incidence of Immediate Improvement in Symptoms	56/78 (72%)	31/34 (91%) *	35/41 (85%) *	66/75 (88%)
Improvement of Symptoms with Bradycardia	75%	87%	80%	84%
Incidence of Total Cure	31/78 (40%) **	23/34 (68%)	22/41 (54%) **	45/75 (60%)
Incidence of No Change in Symptoms	21/78 (27%)	2/34 (6%) *	6/41 (15%) *	8/75 (11%)*
Incidence of Requirement for Post-Op Medications to Treat TN	45/77 (58%) **	8/34 (24%)	21/41 (51%)	29/75 (39%)
Incidence of Post-operative Relapse	8/83 (10%)	4/37 (11%)	4/43 (9%)	8/75 (10%)
Incidence of need to have re- operation	22/78 (28%)	3/34 (9%) *	2/42 (5%) *	5/76 (7%)
Time Until Relapse (Months)	12.1 ± 3.1	10.6 ± 8.5	6.3 ± 1.2	8.4 ± 4.1
Number of Patients Lost to Follow-up (%)	7/82 (9%)	5/37 (14%)	4/43 (9%)	9/80 (11%)

* P < 0.05 compared to BA

** P < 0.05 compared to MVD

All data reported as mean ± SEM

Combined = Microvascular Decompression with or without Rhizotomy

There was a difference in the incidence of postoperative complications (excluding paresthesias) between BA and MVD (Table 4). The incidence of head/face/neck paresthesias was significantly higher in the MVD group (Table 4). Of all remaining complications, only hearing loss occurred with greater frequency in the MVD group compared to those in the BA group (Table 4). The hearing loss noted after MVD was ipsilateral to the surgical field and was transient in all affected patients. There was a higher incidence of CSF leak with MVD but no differences in the incidence of infection or visual changes between the groups (Table 4). All of the complications involving sight were either diplopia or blurred vision which usually resolved in one or two days.

**Table 4:** Post-Operative Complications for Rhizotomy, Balloon Ablation, and MVD

	**BA**	**MVD**	**Rhizotomy**	**Combined**
Overall Complication Rate (%) (*including paresthesias*)	29/83 (35%)	19/37(50%) *	10/43 (23%)	21/80 (26%)
Head/Face/Neck Paresthesias Rate (%)	17/84 (20%)	13/37 (35%)	23/43 (53%)	36/84 (45%) *
Incidence of CSF Leak (%)	0/84 (0%) **	2/37 (5%)	0/43 (0%) *	2/80 (3%)
Incidence of Infection (%)	0/84 (0%)	1/37 (3%)	1/43 (2%)	2/80 (3%)
Incidence of Visual Changes (%)	6/84 (7%)	0/37 (0%)	2/43 (5%)	2/80 (3%) *
Incidence of Hearing Changes (%)	2/84 (2%)	6/37 (16%)*	6/43 (14%)*	12/80 (16%) *

* P < 0.05 compared to BA

** P < 0.05 compared to MVD

Combined = Microvascular Decompression with or without Rhizotomy

## Discussion

Numerous studies have compared different methods of treatment for trigeminal neuralgia but few have compared percutaneous BA of the trigeminal ganglion with posterior fossa MVD [9,15]. Our study is unique in that it compares the long-term outcomes of the two procedures done exclusively by two different surgeons performing the same procedure over a prolonged time period. While the rationale for the decision to have a patient undergo percutaneous BA versus MVD was not always elucidated in the records, the patient's age may have contributed to the decision-making process: patients undergoing balloon compression were significantly older. Finally, patients in the BA group had their disease for a slightly (although not significantly) longer period than patients who underwent MVD. This trend is reported by other studies examining BA procedures and probably reflects a prejudice toward offering this minimally invasive procedure to older patients who typically have more comorbid conditions than their younger counterparts [9,15,16]. However, our data indicated that there was no difference in ASA Physical Status between groups, indicating that co-morbidities may not have contributed to the decision to undergo BA versus MVD in our institution.

Patients undergoing BA also underwent a significantly greater amount of treatments prior to surgery with peripheral nerve blocks as compared to patients undergoing MVD. The 13.1% of our patient population who underwent prior nerve blocks is very similar to the 14.6 percent of patients undergoing nerve blocks reported by Lobato, et al. and may reflect the reluctance of the treating physician to bring older patients to the operating room [17].

Operating times were significantly less with BA compared to microvascular decompression. This was expected since the percutaneous approach is much less invasive and requires much less surgical time compared to the retrosigmoid lateral skull base approach needed for MVD. In a recent analysis by Chen, et al. comparing the same two procedures, our average time of 23 minutes was shorter than their operating time for BA, although the hospital length of stay was equivalent [18]. As would be expected for a more invasive surgical procedure, hospital length of stay was significantly longer in the MVD group.

At the onset of the study, we postulated that the occurrence of intraoperative bradycardia may be suggestive of a favorable outcome in the BA group. The rationale behind this theory was that adequate compression of the trigeminal ganglion required for BA should elicit intra-operative bradycardia through stimulation of the ganglion. Lack of bradycardia may suggest that inadequate ganglion compression occurred with a less than optimal outcome. Our data support this theory. We found that 75% of patients who had bradycardia with BA had some improvement in symptoms. Only 40% of these bradycardic patients had a complete resolution of symptoms. This suggests that bradycardia is not an indicator of successful nerve ablation and that destruction of the ganglion, in many cases, may be incomplete with recurrence of symptoms.

Bradycardia occurred in a majority of the balloon ablation patients and was twice as prevalent when compared to all patients who underwent MVD. Other studies have noted a cardiac depressor response during compression of the trigeminal ganglion [19,20]. Our study found the incidence of bradycardia to be 76%, a value similar to the 70% incidence found by Brown et al [20]. It is interesting to note that the study which demonstrated a 20% vagal response used thermocoagulation while the one where a 70% incidence of bradycardia was observed used microcompression. It may be possible that a compression of the ganglion produces a more severe response than that elicited by thermocoagulation. This response most likely stimulates the efferent arch of the carotid sinus reflex, which ends in the dorsal nucleus of the vagus and produces severe bradycardia or asystole. Finally, one study reported marked tachycardia during ganglion compression [21]. Their report, however, noted initial bradycardia upon entering Meckel's Cave and tachycardia immediately afterward. The explanation for their conflicting findings was light anesthesia and sympathetic stimulation producing tachycardia.

Patient outcomes were very similar in our study compared to reports from the literature [15,18]. MVD showed significantly better immediate relief and total cure rates compared to BA. The higher success rate for microvascular decompression may be due to the fact that this procedure is performed on a discrete lesion or vascular loop which causes the neuralgia and is removed under direct vision. BA neurolysis however, destroys the ganglion but does not affectively remove the cause of the pain. Several studies evaluating outcomes after BA reported total sustained relief between 70–80% [18,19,22]. Our study noted improvements of pain symptoms in 72% of patients, a number similar to the above referenced studies. Immediate improvement in pain symptoms after MVD was 88%, a value very similar to the outcomes reported by other investigations (82–85%) [23,24]. However, long term follow-up showed a total cure rate (i.e. complete resolution of symptoms) of only 60% at a mean interval of 26.5 months, which is lower than reported follow-up success rates of 75% at 1 year, 80% at 38 months, 74% at 5 years, and 64% at 10 years [23-25]. Patients who received an additional rhizotomy had a higher cure rate of 68% still lower than that from other reports. We can offer no reasonable explanation as to why our long-term cure rate is lower than what is reported in literature, except that our definition of cure was 100% resolution of symptoms, whereas other authors may have had a less rigorous definition of "cure", and hence higher successful outcomes.

The incidence of unchanged symptoms after MVD was 11%. While significantly lower than that of the BA group (27%), it is higher than the reported 2% incidence of unchanged symptoms reported by Barker, et al [24]. Other studies however have reported a 7–8% incidence of no change in symptoms after surgery, which closely approximates the incidence in the present study [23,25]. Our relapse rates, defined as a total recurrence of symptoms, were 10% for both groups. This is considerably lower than some studies which reported reoccurrence rates after BA to be as high as 25–30% but equivocal to other studies reporting rates between 9–14% [22,26-28]. Recurrence of TN after MVD has been reported as high as 15.3% in some studies, whereas other studies show a recurrence rate from 6.5–10.2%, again in agreement with our findings [23,29]. Barker, et al., reported several predictive factors for recurrence of TN after MVD, including female sex and a longer preoperative history of TN [24]. Contrary to these findings, we found no significant difference in mean disease length of treatment and the population of patients with total cure versus no cure after MVD. Time until relapse was not significantly different between the BA and MVD groups (12.1 vs 8.4 months, n = 8). These findings are consistent with relapse intervals found for MVD (majority less than 1 year) [30], and for BA (majority within 2 years) [28].

MVD has several related morbidities associated with the procedure. The associated mortality risk is 0.3% with an incidence of neurologic complications reported at 1.7% [31]. Paresthesias are a well-known side effect of this surgery and occurred at a rate of 45% with MVD versus 20% in the BA group. The paresthesia occurrence rate for MVD range from 0.9–4.8% in some papers and 22–36% in others with a higher incidence usually related to concurrence of rhizotomy at the time of MVD [5,8,16]. Our results support the findings of others. Facial paresthesias were much higher in MVD procedures with rhizotomy compared to MVD alone (53% vs 35%). Facial paresthesias for BA surgeries have been reported to be around 15%, a value similar to our findings [8]. Hematoma, mechanical ventilation and facial palsy have also been reported. Other investigators have noted that the three most common complications that occur after MVD are cerebellar injury, CSF leak and hearing loss [24]. None of our patients developed cerebellar injury but 3% developed a CSF leak and 15% of patients undergoing MVD were noted to have hearing loss. No difference was noted in hearing loss if the procedure was also accompanied by rhizotomy. The hearing loss was transient and the short duration of these symptoms suggest conductive hearing loss secondary to fluid in the mastoid air cells after surgery and not direct trauma to the nerve. Some studies reported hearing loss to occur in 24% of all patients while other studies placed hearing lost between 0.8–7.5%, more consistent with our findings [24,29]. Hearing complications may also occur from cerebellar retraction and many have suggested that if cerebellar retraction is necessary, the duration should be brief. The direction of the traction applied should also be perpendicular to the axis of cranial nerve VIII [24]. The surgeon who performed the MVD (DA) did not use cerebellar retraction for these procedures, thus direct trauma to the nerve producing hearing loss was unlikely.

Our study is in agreement with previous comparisons of MVD and trigeminal ganglion BA which show that MVD provides better outcomes with more sustained pain relief and a lower incidence of recurrence [6,10]. The reduced use of medication postoperatively and the lower number of patients that reported no improvement in symptoms after surgery points to the fact that MVD produces a better outcome compared to BA procedures.

Furthermore, our data suggests that a history of prior surgeries for correction of TN is a negative prognostic factor for outcomes with BA surgeries. Even though MVD is more invasive and requires longer hospitalizations, the long-term outcomes favor this procedure.

## Conclusion

In conclusion, we believe that MVD is the best procedure to reduce and eliminate the symptoms of trigeminal neuralgia and recommend this procedure over BA rhizolysis, even in older patients, if their physical status allows them to tolerate a posterior fossa craniotomy.

## List of abbreviations

ASA: American Society of Anesthesiologist; BA: Balloon Ablation; CSF: Cerebrospinal Fluid; MS: Multiple Sclerosis; MVD: Microvascular Decompression; OR: Operating Room; TDR: Trigeminal Depressor Response; TN: Trigeminal Neuralgia.

## Competing interests

The authors declare that they have no competing interests.

## Authors' contributions

WSJ conceived the study and helped draft the manuscript. WB participated in the design of the study and acquisition of the data. KO also was involved with data acquisition and drafting of the manuscript. DA was involved with conceptual design of the study and performed data acquisition, as did JS. EF participated in study design and statistical analysis.
